# Football as an Alternative to Work on the Development of Social Skills in Children with Autism Spectrum Disorder with Level 1

**DOI:** 10.3390/bs11110159

**Published:** 2021-11-19

**Authors:** Jose Maria Lopez-Diaz, Nerea Felgueras Custodio, Inmaculada Garrote Camarena

**Affiliations:** Departamento Ciencias de la Educación, Facultad de CC. Jurídicas y Sociales, Universidad Rey Juan Carlos, 28933 Móstoles, Madrid, Spain; nerea.felgueras@urjc.es (N.F.C.); inmaculada.garrote@urjc.es (I.G.C.)

**Keywords:** ASD, social skills, sport, football, autism spectrum disorder

## Abstract

Given the characteristics of people with Autism Spectrum Disorder, it is evident the difficulties they show in the development of social skills. The scarce participation of people with Autism Spectrum Disorder in group sports can be taken as a reference. The aim of this study was to analyse the impact of football on the development of social skills in children with Autism Spectrum Disorder. In order to measure the sporting impact, it was necessary to implement a football training programme with the intention of evaluating different social skills. Thirteen children participated in the programme, all of them with a diagnosis of Autism Spectrum Disorder and with a severity level of 1. The study was based on a pre-experimental, pre-test/post-test design. Non-parametric tests were used for the statistical analysis, applying the Wilcoxon test. Two specific tools on social skills were used for data collection. The results showed a generalised improvement in the dimensions linked to the social skills assessed. This highlights the possibility of considering group sport as an alternative to be taken into account to work on and enhance social skills in children with Autism Spectrum Disorder.

## 1. Introduction

It is clear that one of the diagnostic criteria for Autism Spectrum Disorder (ASD) is oriented towards the difficulties that people with ASD have in terms of the development of social and communication skills [1]. The problems that arise when interacting derive from the difficulty they have in relating to their peers, as they have problems in understanding the implicit conventional rules of social interaction itself [2]. Add to this the difficulties in interpreting and understanding emotions [3], and the result is continued social isolation. To minimise this, several therapeutic programmes are currently proposed to work on different skills and competences with the aim of improving the quality of life of people with ASD.

Sport has been presented as a therapeutic alternative to work with this group. Sport should be understood as a source of health [4], and as a process of human development where not only physical performance is sought, but also performance in psychological and social functions [5].

The sport of football can be included in the concept of play. Regardless of whether a child has ASD or not, play is of great importance in the development of children as it serves as a necessary support in the developmental process. Play should be seen as a right and an occupational alternative in children’s free time. If increased communication and social relations are to be achieved, the activity must be fun and acquire a cooperative character [6]. Participating in group sports will help the development of social skills due to the contact they must have with other people [7].

Football, being a collective sport, is used in a multitude of programmes as a treatment or therapy due to the number of benefits it provides both physically and socially [8]. Football can be practised in different modalities, that said, it should be pointed out that disability cannot be a barrier that prevents the practice of sport.

However, the high level of sedentary behaviour in children with ASD compared to typically developing groups of children is alarming [9]. Some authors pointed out that children with ASD spend little time on sports leisure [10,11].

In relation to the level of importance of playing sport in the development of social skills, Vonder et al. [12] indicated in their study that those who showed optimal levels in the development of motor skills had less difficulty in socialising. Something similar was reported by Lloyd et al. [13] in their research, as their results revealed that those who had difficulties at the motor level also had a greater deficit in social interaction.

On the other hand, it should be noted that, despite the benefits that football can bring, there are not many records that point to this impact on the group of people with ASD [14]. Although, there are particular cases in which football has served as a supportive and therapeutic alternative to improve the quality of life of participants.

At present, there are football socio-sports schools in charge of educating and instructing people who have some kind of disability or special need with the intention of working towards the inclusion of this group in the world of sport.

Researchers Cei et al. [15] concluded in their football sport programme that it helped to improve motor and psychosocial skills according to the level of severity of ASD. GOL was a football sport project which aimed to work on football skills in adults with intellectual disabilities and ASD [14]. Another project based on a football programme demonstrated that targeted sports training helped to improve motor and social skills in children with autism [16]. In this case, training should be oriented towards a holistic approach in which all sporting, psychological, and social elements of the players are worked on together [17]. The latter is produced by the diversity of situations that occur in the course of the game and by the whole environment that surrounds the sporting context. Therefore, it is important to work on and encourage social skills so that they learn to manage on their own [18]. Moreover, if the necessary support is available, the chance of success increases [19]. Furthermore, the evolution will be more favourable if certain social skills are acquired and if they are able to relate to others.

The aim of this study was to analyse the impact of playing football on the development of social skills in children with ASD. To this end, a sports programme was implemented to measure different social skills in different sports contexts, while at the same time offering the possibility of playing a group sport to those who could not do so because they had an ASD diagnosis.

It should be noted that children with ASD need such programmes to enhance their abilities. However, it should not be forgotten that the design of the tasks must be flexible and individualised [20], as, in this way, the individual needs of each person with ASD can be addressed [5]. The ultimate goal of practising sport is none other than to have a space for social participation where play is the tool that serves to help a child grow and improve as a person, in addition to maintaining an optimal level of health.

## 2. Methodology

### 2.1. Participants

In order to set up the sports project, it was necessary to publicise the programme in schools, associations, and educational institutions so that all children who were interested in practising sport and/or playing football, and who also met the selection profile, could sign up. A multidisciplinary team made up of a physical education teacher and two psychologists was in charge of conducting individual interviews with the families interested in participating. These meetings were used to find out the level of functionality of the children and to observe whether they met the selection profile: age between 6 and 12 years old, and an ASD diagnosis with a severity level of 1.

After completing the selection process, 13 children participated in the football training programme. Their ages ranged from 6 to 10 years (M = 7; SD = 1.42). The total number of participants were boys. Regarding arm and leg dominance, the vast majority of players were right-handed in both cases: 23.1% of the players were left-handed in the arm and 38.5% were left-handed in the legs, while 76.9% of the children were right-handed in the arm and 61.5% were right-handed in the legs. At that time, 53.8% played some kind of sport, although in no case was this practice regulated. The sports practised were divided between judo, karate, swimming, and multisport.

In order to carry out the project, it was necessary to have the supervision of an Ethics Committee. This Committee was responsible for ensuring compliance with existing legislation to protect the rights of participants in the sports programme.

### 2.2. Instruments

As a methodological proposal, a pre-experimental, pre-test/post-test design was carried out, with the intention of measuring the impact that the development of social skills would have on the practice of sport by children with ASD. On the other hand, the Teacher Observation Scale (EOP) by Muñoz, Trianes, Jiménez, Sánchez, and García was adapted from the observation record of the MESSY (The Matson Evaluation of Social Skills in Youngsters) teacher questionnaire. These tools were selected because they were the most suitable for the evaluation process of the different contexts related to social skills.

The self-report Social Interaction Skills Questionnaire (CHIS) was composed of six dimensions: basic social skills, befriending skills, conversational skills, skills related to emotions and feelings, interpersonal problem-solving skills, and adult relationship skills. Fifty-nine items were measured, giving the tool a Cronbach’s alpha reliability of 0.948. Each of the items is divided into five alternatives, which assess the frequency of the child’s behaviour: 1. “never does the behaviour”; 2. “rarely does the behaviour”; 3. “does the behaviour quite often”; 4. “almost always does the behaviour”; and 5. “always does the behaviour”.

The second tool, the Teacher Observation Scale (TOS) was composed of six dimensions: impatience, dominance-aggression, inhibition, helpfulness/cooperation, friendliness, and social responsibility. Forty-two items were measured, giving the tool a Cronbach’s alpha reliability of 0.852. The items were rated on a Likert-type scale with the following values: 1. “almost never”; 2. “sometimes”; 3. “often”; and 4. “always”.

### 2.3. Procedure

In order to work on different social skills with the participants, the football coach programmed 34 one-hour sports training sessions, two days a week. Due to the sporting level of the group, all activities and games were adjusted to simple tasks related to the initiation of the sporting game. It should not be forgotten that play promotes social inclusion and communication skills [21]. In addition, due to the individual character of the training, the tasks themselves were adapted so that each of the children could play according to their individual characteristics.

The diversity of games applied in the training sessions helped to generate diverse sporting and social contexts, hence the importance of working on and developing the players’ social skills. The proposal of games included, on the one hand, purely social tasks, i.e., meetings of players and coach, where the aim was to interact and get to know each other better as a group, and, on the other hand, physical-sporting aspects where the interest was oriented towards playing as a team and learning about the sport. In this case, games based on physical, technical, and tactical elements, such as knowing how to move around the playing space, jump, hit the ball, control it, run with the ball, dribble, defend, and play as a team, helped to improve sporting performance by increasing participation and interaction between the children to play football.

The football training sessions were held in a school sports gymnasium, as well as on the multi-sports court in the playground. In order to facilitate the coexistence of the group, and due to the profile selected for the programme, all training sessions followed a certain structure. The sessions were composed of a warm-up block, a main block, and a cool-down block.

For each block, the games that best adapted to the corresponding sporting characteristics were selected. The warm-up block included all the games that prepare the body for higher intensity sports activities [22]. The main block sustained the greatest workload of the sports training session. In this part, all the games that have a more intense character were re-categorised. In addition, this part grouped together the main objectives of the session. The last block was used for the body to reach a state of relaxation, achieving the same state as at the beginning of the session [22].

To conduct the study, it was necessary to involve three independent external judges with psychologist profiles. Before the start of the programme, the three judges were trained beforehand in order to understand the dynamics of the sessions. This made it possible for the three judges to be on equal footing and with the same level of knowledge, and thus able to apply the same criteria in the evaluation of the children. This methodology based on inter-judge consistency helped to analyse whether the opinion and the results coincided or were close in terms of the evaluation of the results.

### 2.4. Statistical Analysis

Data analysis was carried out with the SPSS version 22 statistical programme. The results of the socio-demographic data were obtained through a descriptive analysis process. On the other hand, in order to know the impact that football training had had on children with ASD, a statistical analysis based on non-parametric tests was applied using the Wilcoxon test, with the aim of comparing the pre- and post-results of the same group, at two different time points. To measure inter-rater consistency, it was necessary to calculate Krippendorff’s alpha for both tools. On the Self-Report Social Interaction Skills Questionnaire (CHIS) the result obtained was 0.8101, while on the Teacher Observation Scale (EOP) the result was 0.867.

## 3. Results

The results are divided into two blocks: the first one covers socio-demographic results, while the second block focuses on results related to children’s social skills.

### 3.1. Results on Socio-Demographic Data

Of all the children who participated in the sports programme, 53.84% were practising a sport at the time, with swimming being the individual sport most practised. On the other hand, 38.46% indicated that before the start of the programme they had played sport, although at that time they had not kept it up over time. The sports they had practised were karate, swimming, roller skating, multisports and football, both individually and informally.

### 3.2. Results Regarding the Social Skills of the Participants

Regarding the dimension related to basic social skills (Table 1), it can be noted that there was a general improvement in the group. All items improved their results with respect to the initial level, although five of them presented values below the average. In addition, the items of laughing with others when appropriate (*p* ≤ 0.005), showing polite behaviour (*p* ≤ 0.005), and introducing oneself to others when necessary (*p* ≤ 0.005) were significant.

Regarding the dimension of skills to make friends (Table 2), it is worth noting that all the results of all the items improved with respect to the initial level, although in no case were these improvements significant.

On the other hand, in the conversational skills dimension (Table 3), six of the ten items scored below the mean at the start of the sport programme. Despite the variability of changes in scores, these same six items still ended up with scores below the mean. In addition, three items showed worse scores than at baseline. However, in no case were the changes in improvement or regression significant.

Regarding the dimension on skills related to emotions and feelings (Table 4), it should be noted that all items improved their results, and in some cases with a difference of one point or more with respect to the initial level. In addition, the item related to defending and claiming their rights showed a significant improvement (*p* ≤ 0.005).

Regarding the dimension on interpersonal problem-solving skills (Table 5), eight of the items showed improvements in their scores with respect to the initial level. The item on the ability to seek and generate possible solutions when a problem arises and the item on the ability to identify conflicts that arise when interacting with other children showed a decrease in mean scores compared to the beginning of the programme. On the other hand, the item on anticipating the consequences of their own actions when a problem arises (*p* ≤ 0.005), the item on anticipating the consequences of the actions of others when a problem arises (*p* ≤ 0.005), and the item on identifying the causes that led to the problem (*p* ≤ 0.005) showed significant improvements.

On the other hand, eight items of the dimension on relationship skills with adults (Table 6) improved their results with respect to the beginning of the programme. Moreover, in the case of praising and saying positive things to adults, the improvement of the result was significant (*p* ≤ 0.005). It should be noted that the score for the item “resolving interpersonal conflicts with adults” did not undergo any variation, while the item “when having a problem with an adult, puts himself in the place of the adult to solve it” showed a regression in the result.

With regard to the impatience dimension (Table 7), six items improved their scores from baseline. The item “being impulsive” did not show any variation in score, while the item “constantly changing activity” suffered a regression in score from the initial level.

In the dimension on dominance and aggressiveness (Table 8), five items showed an improvement in scores from baseline. The items “teasing or playing practical jokes” and “protesting when told to do something” suffered a drop in scores, while the remaining item “making trouble when with others” did not show any variation.

Regarding the inhibition dimension (Table 9), more than half of the items improved their scores compared to the beginning of the programme. The items “being embarrassed when the coach corrects him/her” and “conforming when he/she is excluded from the game” showed no variation in the results, while the item “rarely looks directly into the eyes of his/her interlocutor” showed a regression in the score.

Four items of the helping and cooperating dimension (Table 10) showed improvements in scores compared to the initial level. The remaining two items, “try to take into account the views of others when problems arise” and “participate smoothly in different group activities independently of group mates” showed no change in scores.

All items of the friendship dimension (Table 11) improved their scores with respect to the start of the programme. In addition, the improvement of the item “tolerates teasing or provocation well” was significant (*p* ≤ 0.005).

As for the dimension on social responsibility (Table 12), six items showed improvements in their scores compared to the initial level. However, the item “being sincere and honest” did not show any variation in the score.

## 4. Discussion

At the end of the sports training programme, the improvement in the development of the participants’ social skills was evident, highlighting the importance and benefit of playing sport for children with ASD. However, as expected, the improvement in social skills was not regular and constant in each of the skills assessed, nor was the individual performance of the players. The interviews were used to find out the sporting and social level from which the participants started and, thus, to be able to begin designing the sports training sessions. It was important to know whether they had previously had contact with any sport and what type of sport. In this case, the vast majority had played individual sports. However, the proposal to propose a collective sport was due to the number of benefits it could bring both physically and socially [8]. Being aware that none of the participants had ever formally played football, and that the little practice they had had was limited, it was determined that all the children were at a stage of sport initiation. In this case, the sport training design was the same for all of them.

With regard to specific tools that assess social skills, it should be noted that there is no tool that measures social skills in children with ASD. Taking into account the difficulty of carrying out any type of intervention with this group, it is more complex to generalise the results [23].

Due to this situation and the type of sporting activity chosen, it was necessary to select two questionnaires that could measure different social dimensions. Care must also be taken when interpreting the results, as the limitations that children present at a social level due to their diagnosis of ASD must be taken into account.

However, the results obtained project an improvement in social skills, as the behaviour and conduct shown at the end of the programme was positive in most cases. It is worth noting that many of the items assessed were interrelated. This meant that, in many of the contexts, different items could be measured simultaneously. However, in some cases this was not achieved due to the specificity of the items, so it was necessary to observe this cross-sectionally within the games themselves.

All training sessions followed a certain structure, as planned physical activity favours social relationships [24]. Two specific tasks were designed to better understand the social and personal aspects of the children. Moreover, these tasks were repeated throughout the training sessions, which helped to generate routines.

Although during the training sessions a cordial treatment between the players was achieved, at the end of the programme some of the social relationships were affected. This situation contributed to the creation of small groups due to common preferences and tastes. As pointed out by Martín [25], many of the difficulties presented by children with ASD may be due to unusual interests that they do not share with their peers.

It is important to note that, at the beginning of the programme, a large part of the group had serious difficulties in processing information [26] and interpreting the context of the conversation. As Miguel [27] points out, people with ASD have a great deficit in the interpretation of emotions and in the understanding of facial expressions [3]. As a result, some children showed behavioural disturbances, impatient behaviour, irritability, and difficulties in concentrating on play.

However, after having learned to respect the rules of the conversation (taking turns, knowing how to maintain a dialogue, showing an appropriate attitude, etc.), there was a shift from complying with the rules to doing so only at certain times because the children’s main motivation was to play. Here it was possible to observe the impulsive and impatient behaviour of some of them.

On the other hand, it was shown that the participants were not able to take the initiative, so it was necessary for the trainer to find alternatives for them to do so.

All activities in which the player had freedom of movement and decision-making provoked anxiety levels and disruptive behaviours in some children. Muñoz [28] considers that this type of behaviour is due to a communication intention. Therefore, in this case, children may have displayed this type of behaviour to compensate for their difficulties, and as a way of expressing their needs [28].

Mental fatigue was another factor affecting mood, information processing and interpretation, and behaviour [29]. Attitudes related to dominance and aggressiveness were observed here.

As for the dimension on interpersonal problem-solving skills, there was little change during the programme. Children did not show initiative and ability to resolve conflict situations due to difficulty in understanding their own and others’ feelings and motivations [30]. To minimise disruptive behaviours, intervention by the coach was necessary. In this case, the solution was to spend more time reflecting on the behaviour displayed in order to avoid possible repetition of inappropriate behaviour. In some cases, some children were able to identify personal conflicts with their peers, although the level of response shown to resolve them was very limited.

On the other hand, it should be noted that not all participants responded in the same way and with the same intensity. A general improvement was observed, although there were cases where the circumstances of the sessions were not favourable enough to really measure the impact of the actions at a social level. In order to work on some social skills, it might be better to propose specific workshops in addition to sport.

Finally, taking into account the characteristics of the research, it cannot be stated that the results can be conclusive, although it does initiate an alternative path to the therapies already used to work on social skills with people with ASD. The lack of a control group in the research means that the results obtained cannot be more decisive. In addition, the small number of children who participated in the study may be another drawback in the generalisation of results. Likewise, the fact that there are no specific validated questionnaires to measure the social behaviour of children with ASD creates an added difficulty in evaluating and obtaining more precise data. Therefore, it is important to remain cautious about the interpretation of the results obtained. Finally, in order to be able to evaluate whether the duration of the programme has been sufficient, it would be advisable to carry out periodic follow-ups to check whether or not the improvements obtained are maintained over time.

## 5. Conclusions

The sports training programme can be considered to have been positive. Any learning or modification of social skills that took place during the training sessions required a complex process. Not all participants responded in the same way and with the same intensity. It should be noted that an improvement was observed in many of the items of the different dimensions evaluated, however, there were others that, due to the circumstances of the sessions, meant it was not possible to work on them enough to be able to really evaluate the impact due to the infrequency that occurred in the actions. In this case, the best way to work on certain social skills is to accompany sport practice with specific complementary workshops, so that controlled scenarios can be established with the aim of evaluating and working on these specific skills.

During the development of the work, the aim was to show the relationship between ASD and sport. It is important to point out that if children with ASD play football following targeted training, there may be a differential improvement in the performance of social skills. In this case, it is essential that coaches follow specific methods adjusted to the sporting levels of the players. Moreover, as it is a group sport, social situations will be generated, such as, for example, being exposed to a group or resolving conflicts that arise in coexistence, which will favour an improvement in the acquisition and performance of social skills. The results obtained confirm that the programme has proved to be efficient and healthy for children with ASD, but in some cases not with the desired intensity.

On the other hand, this research work allows us to offer different socialisation alternatives in which sport plays a leading role due to its therapeutic value and the potential it has as a game for children. This type of programme can help to promote healthy lifestyles and, therefore, improve the quality of life of its participants. Likewise, it should not be forgotten that the purpose of the training should be to educate in values and to accompany the individual growth of the children. Children with ASD evolve favourably if they have acquired certain social skills, if they learn to interact with other people, if they learn to follow rules and instructions, and if they are able to organise their activities following a structure determined by themselves.

## Figures and Tables

**Table 1:** Basic social skills.

	PRE		POS		Z	Sig. *
	M	SD	M	SD		
Greets correctly	3	0.8	3.7	0.9	−2.24	0.041
Responds well when addressed by other children	1	0	2.1	1	−2.65	0.009
Laughs with others when appropriate	2.8	0.8	3.7	0.9	−2.82	0.005
Smiles at others in appropriate situations	3.1	1	3.4	1	−1.83	0.261
Responds well when greeted by others	2.1	0.9	2.7	0.8	−1.91	0.082
Asks for favours when needed	1.5	0.7	2	1	−1.95	0.07
Shows polite behaviours (e.g., says thank you or apologises)	1.4	0.6	2.8	0.9	−2.96	0.003
Introduces himself/herself to others when necessary	2.9	0.9	3.7	1.1	−2.95	0.003
Does favours for others	1.1	0.2	1.9	1.2	−2.24	0.026

Note: Maximum score = 5; minimum score = 1; * significant values *p* ≤ 0.005; PRE = pre-test; POS = post-test; M = mean; SD = standard deviation.

**Table 2:** Skills for making friends.

	PRE		POS		Z	Sig. *
	M	SD	M	SD		
Asks for help when needed	2	1	2.9	1.1	−2.53	0.017
Praises, says positive things to other people	2.1	0.9	2.5	1.1	−1.88	0.079
Responds appropriately when invited to play by another child	2.6	0.8	3.5	0.9	−2.46	0.025
Shares own things with other children	3	1	3.6	0.8	−1.48	0.247
Cooperates with other children in various activities and games	2.8	0.9	3.3	0.9	−1.99	0.067
Helps other children on different occasions	2.8	0.9	3.2	0.9	−1.13	0.275
Responds well when praised by others	2.6	0.9	3.5	0.9	−2.25	0.04
Joins in with other children who are playing	1	0	1.4	0.7	−1.8	0.073
Initiates play with other children	1.4	0.6	1.9	0.4	−2.15	0.035
Responds appropriately when another child wants to join in play	2.9	0.8	3.3	1	−1.38	0.195

Note: Maximum score = 5; minimum score = 1; * significant values *p* ≤ 0.005; PRE = pre-test; POS = post-test; M = mean; SD = standard deviation.

**Table 3:** Conversational skills.

	PRE		POS		Z	Sig. *
	M	SD	M	SD		
Responds well when another child wants to enter into a conversation with others	3.2	0.8	3.9	1.1	−2.24	0.03
Responds well when the people with whom he/she is talking end the conversation	2.6	1	3.5	1	−2.43	0.02
When conversing, listens to what is said, responds to questions, and expresses thoughts and feelings	2.5	0.8	3.1	0.9	−1.97	0.212
When chatting with other children, ends the conversation in an appropriate way	1.9	0.8	1.2	0.4	−1.85	0.338
Initiates conversations with other children	1.3	0.6	1	0	−1.83	0.073
When having a conversation with other people, participates actively	1.6	0.8	2.1	0.8	−1.8	0.108
When conversing with a group of children, participates according to established norms	1.2	0.4	1	0	−1.14	0.264
When having a group conversation, intervenes when appropriate and does so in a correct manner	1.6	0.7	2.1	0.8	−1.77	0.126
Joins in conversation with other children	1	0	1.1	0.2	−0.45	0.727
Responds appropriately when another child wants to initiate a conversation with him/her	2.6	1	3.2	1	−1.72	0.212

Note: Maximum score = 5; minimum score = 1; * significant values *p* ≤ 0.005; PRE = pre-test; POS = post-test; M = mean; SD = standard deviation.

**Table 4:** Skills related to emotions and feelings.

	PRE		POS		Z	Sig. *
	M	SD	M	SD		
Responds well to the emotions and pleasurable feelings of others	3.3	0.9	4.4	0.7	−2.73	0.013
Defends and claims his/her rights	1	0	2.2	0.7	−3.06	0.003
Expresses and defends his/her opinions appropriately	2.1	0.9	2.7	0.9	−2	0.059
Says positive things to him/herself	2.6	1	3.9	1	−2.86	0.006
Expresses pleasant emotions and feelings appropriately	2.5	0.9	3	0.9	−1.91	0.086
Expresses unpleasant emotions and feelings appropriately	2.3	1	3.1	0.7	−2.17	0.055
Responds well to the emotions and unpleasant feelings of others	2.7	0.8	3.1	0.6	−1.46	0.273
Expresses positive things about themselves to others	1.5	0.7	2.9	0.9	−2.7	0.008
Responds well to other people’s defence of their rights	1.8	0.9	2.9	0.9	−2.25	0.054
Expresses disagreement and disagreement with others	3	0	3.3	0.8	−1.19	0.325

Note: Maximum score = 5; minimum score = 1; * significant values *p* ≤ 0.005; PRE = pre-test; POS = post-test; M = mean; SD = standard deviation.

**Table 5:** Interpersonal problem-solving skills.

	PRE		POS		Z	Sig. *
	M	SD	M	SD		
When faced with a problem, evaluates the results obtained after trying to solve it	2.9	0.9	3.2	0.7	−1.03	0.415
When faced with a problem with other children, chooses an effective and fair alternative solution	2	0.8	2.6	0.8	−1.88	0.064
When they have a problem, they put themselves in the other’s shoes by looking for alternative solutions	3	1	3.7	1.1	−2.28	0.032
When faced with a conflict, plans the implementation of the chosen solution	2.4	1	3	1	−1.69	0.14
When they have a problem, they anticipate the likely consequences of their own actions	1.8	0.6	3.2	0.7	−3.19	0.002
When faced with a problem, anticipates the likely consequences of the actions of others	1.4	0.5	2.5	0.5	−2.99	0.003
When faced with a problem, seeks and generates several possible solutions	1.1	0.3	1	0	−1.14	0.264
Identifies conflicts that arise when interacting with other children	1.4	0.7	1	0	−1.82	0.071
When trying to solve a problem with other children, evaluates possible solutions in order to choose the best one	3.2	0.7	3.6	0.9	−1.34	0.317
When faced with a problem, identifies the causes of the problem	3.8	0.5	5	0	−3.36	0.001

Note: Maximum score = 5; minimum score = 1; * significant values *p* ≤ 0.005; PRE = pre-test; POS = post-test; M = mean; SD = standard deviation.

**Table 6:** Relationship skills with adults.

	PRE		POS		Z	Sig. *
	M	SD	M	SD		
Resolves interpersonal conflicts with adults	2.8	0.8	2.8	0.8	−1.1	0.427
Responds appropriately to suggestions and demands of adults	3.4	0.7	4.2	0.8	−2.72	0.01
Praises and says positive and nice things to adults	1	0	2.2	0.9	−2.84	0.005
Responds well when adults address him/her kindly	2.9	0.8	3.2	1.2	−1	0.373
When he/she has a problem with an adult, puts him/herself in the adult’s shoes to solve it	3	0.9	2.8	1	−0.66	0.513
Has conversations with adults	2.8	0.8	3.4	0.7	−1.75	0.123
Makes suggestions and complaints to adults	2.5	0.8	3.1	0.9	−2.16	0.038
Initiates and ends conversations with adults	2.1	0.9	2.6	1.1	−1.5	0.217
When praising and complimenting adults, is sincere and honest	1.9	0.8	2.8	1	−2.17	0.134
When interacting with adults, is sincere and honest	2	1.2	2.5	1	−1.95	0.142

Note: Maximum score = 5; minimum score = 1; * significant values *p* ≤ 0.005; PRE = pre-test; POS = post-test; M = mean; SD = standard deviation.

**Table 7:** Impatience.

	PRE		POS		Z	Sig. *
	M	SD	M	SD		
Insists and gets annoyed when refused something he/she has asked for	2.7	1	3	0.9	−1.43	0.172
Rushes through tasks or leaves them unfinished	2.8	1.2	3.2	0.9	−1.28	0.208
Keeps his/her turn	2.7	1.1	3.3	0.7	−1.91	0.056
Constantly demands the attention of the trainer	2.8	1.2	2.9	1	−0.57	0.596
Is impulsive in behaviour	3	1	3	0.8	−0.33	0.772
Constantly changes activities	3.1	1.1	3	0.9	−0.97	0.390
Is easily frustrated	2.9	1	3.1	1	−0.65	0.608
Frequently leaves his/her seat claiming that he/she needs something to continue his/her task	3.1	1	3.5	0.7	−1.67	0.109

Note: Maximum score = 4; minimum score = 1; * significant values *p* ≤ 0.005; PRE = pre-test; POS = post-test; M = mean; SD = standard deviation.

**Table 8:** Dominance-aggressiveness.

	PRE		POS		Z	Sig. *
	M	SD	M	SD		
Frequently gets into fights	3.5	0.9	3.7	0.7	−1.31	0.263
Reacts violently if an object is taken away from them	3.2	1.1	3.7	0.5	−2.1	0.039
Teases or plays practical jokes on peers	3.9	0.3	3.5	0.7	−1.71	0.124
When with others, is the trouble maker	3.3	0.9	3.3	0.8	−0.61	0.559
Enjoys witnessing aggressive and violent actions	3.7	0.8	3.9	0.5	−1.35	0.192
Protests in word or deed when ordered to do something	4	0	3.4	0.7	−2.39	0.02
Is sometimes compliant and sometimes abrupt and provocative	3.3	0.9	3.5	0.6	−1.31	0.201
Becomes disproportionately angry with peers if they play a joke on him/her	3.6	0.7	3.9	0.4	−1.61	0.212

Note: Maximum score = 4; minimum score = 1; * significant values *p* ≤ 0.005; PRE = pre-test; POS = post-test; M = mean; SD = standard deviation.

**Table 9:** Inhibition.

	PRE		POS		Z	Sig. *
	M	SD	M	SD		
Avoids speaking; even when others ask him/her questions he/she finds it difficult to respond	2.9	0.8	3.5	0.8	−2.39	0.017
Is disproportionately embarrassed when coach corrects him/her	4	0	4	0	0	1
In group tasks, remains isolated or keeps at a distance	3.1	0.8	3.4	0.9	−1.69	0.144
If others do something to him or exclude him from the game, he is compliant	2.5	0.8	2.5	0.8	−0.43	0.687
Is seen more often playing alone than with peers	3	0.7	3.1	1	−0.84	0.404
Rarely looks directly into the eyes of his interlocutor	2	0.8	1.8	0.7	−0.69	0.637
Is shy	2.6	0.8	3.5	0.8	−2.47	0.016
Finds it difficult to relate to others	2.5	0.7	3	0.7	−1.83	0.081

Note: Maximum score = 4; minimum score = 1; * significant values *p* ≤ 0.005; PRE = pre-test; POS = post-test; M = mean; SD = standard deviation.

**Table 10:** Assistance/cooperation.

	PRE		POS		Z	Sig. *
	M	SD	M	SD		
Tends to help peers when needed	1.2	0.4	1.6	0.8	−1.77	0.092
Volunteers for class activities	1.6	0.8	1.7	0.8	−0.78	0.47
Tends to encourage participation in group work	1.3	0.4	1.7	0.9	−1.85	0.081
Tries to take others’ views into account when problems arise in class	1	0	1	0	0	1
Accepts suggestions and corrections from others when doing any kind of group work	1	0	2	0.8	−2.74	0.007
Participates in different group activities independently of group mates without any problem	2.9	0.4	2.9	1	−0.68	0.561

Note: Maximum score = 4; minimum score = 1; * significant values *p* ≤ 0.005; PRE = pre-test; POS = post-test; M = mean; SD = standard deviation.

**Table 11:** Friendship.

	PRE		POS		Z	Sig. *
	M	SD	M	SD		
He/she is kind	2.7	0.8	3	1	−1.5	0.287
Speaks well of other children	1	0	2	0.9	−2.6	0.015
Tolerates teasing or provocation well	1.8	0.6	3.4	0.8	−3.01	0.003
Is a nice boy/girl	2.9	0.9	3.2	0.9	−1.32	0.274
Children like to hang out with him/her	2.6	0.6	3	1	−1.33	0.372

Note: Maximum score = 4; minimum score = 1; * significant values *p* ≤ 0.005; PRE = pre-test; POS = post-test; M = mean; SD = standard deviation.

**Table 12:** Social responsibility.

	PRE		POS		Z	Sig. *
	M	SD	M	SD		
Usually complies with class rules	2.6	1	3	0.8	−1.76	0.13
Is involved in the smooth running of the class	2.4	1.1	2.7	1	−0.95	0.366
Is sincere and honest	4	0	4	0	0	1
Shows politeness in dealing with others	2.7	0.8	3.2	0.9	−2.09	0.093
Feels an obligation to help a child who is in difficulty	1	0	1.9	0.8	−2.8	0.006
Can be given tasks with the confidence that he/she will carry them out as they are done	2	0.9	2.5	1	−2.06	0.04
If no one volunteers to do a common task, he/she finally agrees to do it himself/herself	1.9	0.8	2	0.9	−0.78	0.471

Note: Maximum score = 4; minimum score = 1; * significant values *p* ≤ 0.005; PRE = pre-test; POS = post-test; M = mean; SD = standard deviation.

## Data Availability

The data is available from the corresponding author.
